# Amplicon-Based RNA Interference Targeting V2 Gene of *Cotton Leaf Curl Kokhran Virus*-Burewala Strain Can Provide Resistance in Transgenic Cotton Plants

**DOI:** 10.1007/s12033-016-9980-8

**Published:** 2016-10-18

**Authors:** Aneela Yasmeen, Sarfraz Kiani, Afshan Butt, Abdul Qayyum Rao, Faheem Akram, Aftab Ahmad, Idrees Ahmad Nasir, Tayyab Husnain, Shahid Mansoor, Imran Amin, Shaheen Aftab, Muhammad Zubair, Muhammad Nouman Tahir, Sohail Akhtar, Jodi Scheffler, Brian Scheffler

**Affiliations:** 1 Center of Excellence in Molecular Biology (CEMB), University of the Punjab, Lahore, Pakistan; 2 National Institute of Biotechnology and Genetic Engineering, Faisalabad, Pakistan; 3 Jamie Whitten Delta States Research Center, USDA, Stoneville, MS 38776 USA

**Keywords:** Gene transformation, siRNA, CLCuD, Begomovirus, V2 ORF, Knockdown

## Abstract

The conserved coat or V2 gene of begomoviruses is responsible for viral movement in the plant cells. RNAi technology was used to silence V2 gene for resistance against these viruses in transgenic plants. The transformation of the RNAi-based gene construct targeting V2 gene of CLCuKoV-Bur, cloned under 35S promoter, was done in two elite cotton varieties MNH-786 and VH-289 using shoot apex cut method of gene transformation. The transformation efficiency was found to be 3.75 and 2.88 % in MNH-786 and VH-289, respectively. Confirmation of successful transformation was done through PCR in *T*_0_, *T*_1_, and *T*_2_ generations using gene-specific primers. Transgenic cotton plants were categorized on the basis of the virus disease index in *T*_1_ generation. Copy number and transgene location were observed using FISH and karyotyping in *T*_2_ generation which confirmed random integration of V2 RNAi amplicon at chromosome 6 and 16. Real-time quantitative PCR analyses of promising transgenic lines showed low virus titer compared to wild-type control plants upon challenging them with viruliferous whiteflies in a contained environment. From the results, it was concluded that amplicon V2 RNAi construct was able to limit virus replication and can be used to control CLCuV in the field.

## Introduction

The agriculture sector is of utmost importance for Pakistan’s economy as it contributes 21.4 % of the overall GDP. Among major crops, only cotton shares 1.2 % of the country’s overall GDP and contributes more than 60 % of its foreign exchange. Cotton leaf curl disease (CLCuD) is a major threat to the production of cotton in Pakistan [1–3]. CLCuD remained a sporadic nuisance before 1986, but in the subsequent years, it rapidly spread across the cotton growing areas of Pakistan and became an epidemic in 1991–2 causing heavy yield losses during those years. During the late 1990s, resistant cotton varieties were introduced and thus losses due to this disease reduced [4]. However, in the year 2001–2, in the Burewala region of Punjab province, resistant cotton varieties began to show typical symptoms of CLCuD. This was an indication of a second epidemic and referred to as *Cotton leaf curl Burewala virus* which is now known as *Cotton leaf curl Kokhran virus*-Burewala strain (CLCuKoV-Bur). This virus has spread and infection is now found in most cotton growing areas in Pakistan [5, 6].

Typical symptoms of CLCuD include thickening, darkening and swelling of veins, upward or downward curling of leaves, and enations (cup-shaped laminar outgrowths on the undersides of leaves) [7–9]. The CLCuD is caused by a complex consisting of several monopartite begomoviruses (family *Geminiviridae*) that essentially require a satellite molecule known as cotton leaf curl Multan beta satellite (CLCuMB). CLCuMB is entirely dependent on the helper begomovirus for its replication and encapsulation, while it acts as a pathogenicity determinant and encodes protein βC1 that can overcome host defense responses [10]. It has been shown that CLCuKoV-Bur is a recombinant molecule derived from two previously reported viruses i.e., *Cotton leaf curl Multan virus* and *Cotton leaf curl Kokhran virus* [6]. Similarly, the beta satellite associated with CLCuKoV is a recombinant of CLCuMB and Tomato leaf curl beta satellite [11]. Also associated with the disease is another self-replicating component referred to as an alpha satellite. The role of the alpha satellite is not fully known; however, the rep protein of an alpha satellite has been shown to be a suppressor of gene silencing [6]. These viral components are transmitted to the plant by whitefly (*Bemisia tabaci*) [12].

To date, no natural resistance to immunity is available in cotton against CLCuD. Besides conventional methods, the use of biotechnological approaches can be a possible solution to this problem, based on RNA interference (RNAi) in varieties with an agronomical CLCuD-tolerant background [13–15]. RNAi (or gene silencing) is a homology based down-regulation/silencing of genes mechanism which is evolutionary conserved and works in a sequence-specific manner. Begomoviruses can be targeted by gene silencing both at the transcriptional level [transcriptional gene silencing (TGS)], which results in methylation of viral DNA, and the post-transcriptional level [post-transcriptional gene silencing (PTGS)], which results in degradation of viral transcripts. RNAi is always triggered by a dsRNA that is cleaved into short interfering siRNAs by an RNase referred to as DICER-like. The siRNAs then guide sequence-specific silencing. For PTGS, siRNAs are incorporated into an enzyme complex, the RNA-induced silencing complex which degrades homologous mRNAs [16–19]. Resistance against viruses in plants can be obtained by inducing RNAi in plants through the introduction of a sequence homologous to the virus in the form of a hairpin (hp) [20]. One of the advantages of RNAi is the silencing signal which is not limited to individual cells, but can spread to neighboring cells and more distant tissues [21, 22].

RNA interference is an emerging technology for developing insect resistance genes. Many transgenic RNAi-based genetically modified (GM) plants, targeting insects have been developed, including GM corn, GM rice, and GM cotton, and additional GM crops are in the process of development. Various researchers have tried to use RNAi technology to obtain resistance against geminiviruses [23–26]. Recently, RNAi-based resistance has been successfully applied in beans against *Bean golden mosaic virus* in Brazil [27, 28].

In this study, two elite cotton varieties i.e., MNH-786 and VH-289 were transformed with an amplicon RNAi construct against V2 gene of CLCuKoV-Bur. The philosophy of silencing of this gene was to restrict virus movement and further spread. Similar efforts were made for transforming Indian cotton varieties with RNAi gene constructs targeting V2 and Intergenic region (IR) [15, 29].

## Materials and Methods

### Gene Construct

Two hundred and forty Nucleotides from V2 of *Cotton leaf curl Kokhran virus*-*Burewala* (CLCuKoV-Bur; Accession No. AM421522) were taken to make hairpin construct synthetically. Both sense and antisense sequences of V2 are separated by 115 nucleotides from an intron of *Mungbean yellow mosaic India virus* (MYMIV), Accession No. FM202439, to induce hairpin. The IR of CLCuMuV (Accession No. AY312430) was used, which presumably contains both the Rep promoter and the viral origin of replication. The IR, which is used for higher expression of siRNAs, consists of 287 nt [30]. The only way this amplicon construct can work is by having two IRs for replicational release by the Rep of the infecting virus. However, to make the construct as a defective interfering molecule, 247 nt from Poly A of Cotton leaf curl Multan alpha satellite (CLCuMuA), accession no. AJ132344, were added after the hairpin construct. As a result, the total size of the construct would be 1.479 kb which is approximately equal to the defective molecules of DNA A of begomoviruses.

**Fig. 1:**
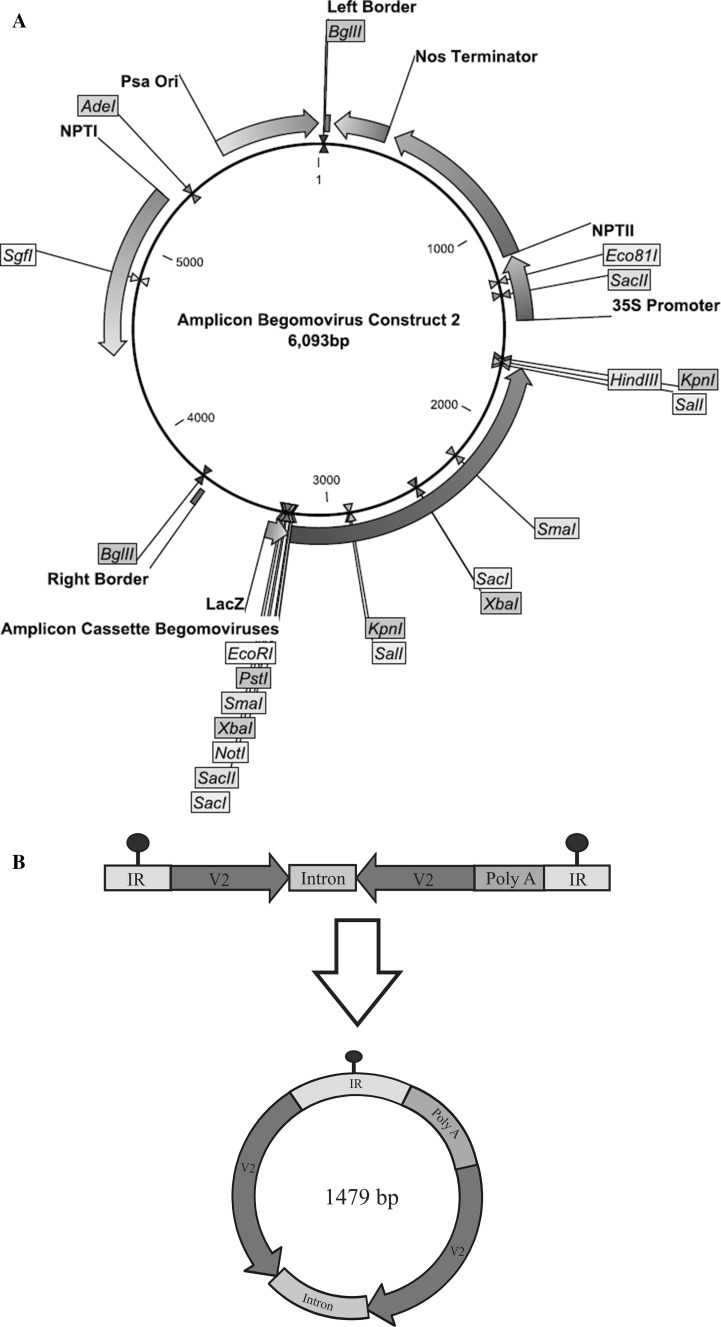
Vector carrying Amplicon-Based RNAi gene targeting Begomoviruses

The construct contains two IRs that enable the construct to be replicationally released from the plant genome, circularized, and replicated by the Rep of the infecting begomovirus. Thus, the construct will constitutively express siRNA derived from V2 gene and will replicate as an episome upon virus infection. The construct was initially cloned in pTZ57R/T vector and subsequently; the fragment was directionally cloned at *Hind* III and *EcoR*I sites into the binary vector pGreen0029. This construct will be called as amplicon V2 RNAi in the manuscript (Fig. 1a, b).

### Primer Designing

Internal primers were designed for the construct (primer details are given in Table 1).

**Table 1:** Internal primer details for gene amplification of RNAi gene construct and real-time PCR

Name	Primer sequence	Length	Tm	Product size (bp)
C2-int (*L*)	TCATAATCTAAACCAAACAGGGAAA	25	60.11	**540**
C2-int (*R*)	TTACAATCAGGTCCTTCAGCAAA	23	60.99	
ClCuV Burewala (*F*)	CGAAAGAAGAAGGAGAAAAA	23	53.0	**203**
ClCuV Burewala (*R*)	AGCAAGAGGAGGACAGCAGA	20	59.4	
ClCuV Multan beta (*F*)	GTTCCGCTGGTTGTCATTTC	20	55.4	**268**
ClCuV Multan beta (*R*)	CCTCTTCAGTTCCGTTTTTC	21	54.6	

Bold values are the important facts and figures about results

### Confirmation of Amplicon-Based Begomovirus Construct

The amplicon V2 RNAi construct was confirmed through amplification using gene-specific primers. The construct was also confirmed, using plasmid as template, through restriction digestion analysis. Furthermore, confirmation of successful electroporation in *Agrobacterium* was also done through colony PCR using gene-specific primers.

### Plant Material

On request, seeds of cotton varieties MNH 786 and VH 289 were provided by the Cotton Research Institute, Multan and Cotton Research Institute, Vehari, respectively.

### Plant Transformation

**Fig. 2:**
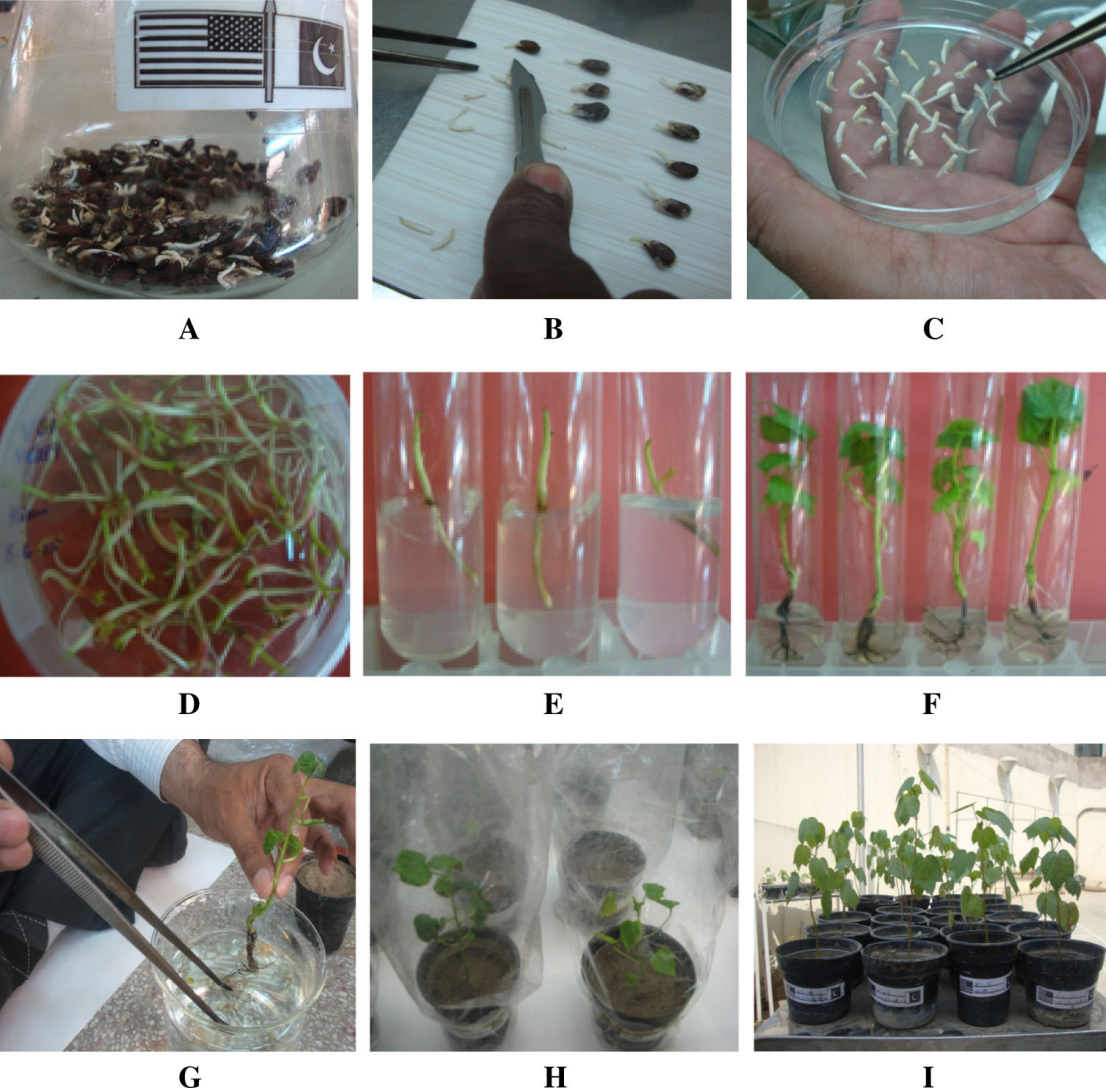
Steps from embryo transformation to shifting of plants in soil pots **a** Germination of Embryos in sterilized flasks, **b** Embryos isolation from seeds, **c** *Agrobacterium*-treated embryos are co-cultivated on MS medium, **d** *Agrobacterium*-treated embryos growing on MS medium (4 days), **e** Inoculation of embryos in test tubes containing MS media with kanamycin selection, **f** Roots developing in MS rooting medium, **g** Transgenic plants being shifted in soil pots, **h** putative transgenic plants in soil pots covered with polythene bags, **i** Acclimatization of putative transgenic plants

**Fig. 3:**
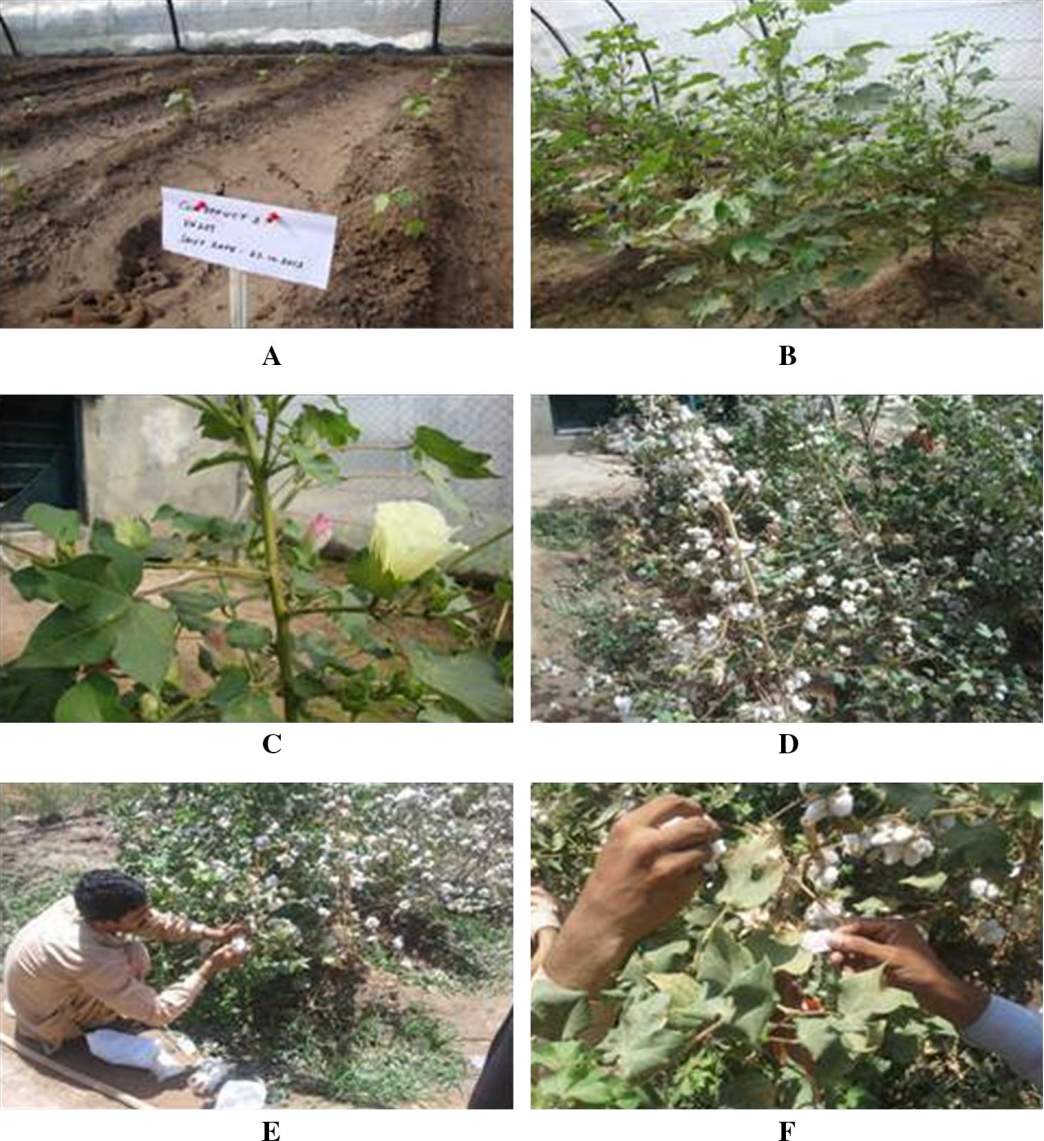
Putative (*T*_0_) plants in field **a** Newly shifted *T*_0_ transgenic plants, **b** *T*_0_ plants thriving in the field, **c** selfing of bolls to self-cross the seeds, **d** *T*_0_ bolls at maturity, **e** and **f** Picking of *T*_0_ bolls

Amplicon V2 RNAi was transformed in embryos via *Agrobacterium* transformation through the Embryo shoot apex cut method as described by Rao et al. [31, 32]. A total of 14200 embryos were used in transformation experiments from which 4000 were of control. The plantlets were given kanamycin selection (100 µg/ml) in the shoot development medium (Murashige and Skoog medium MS: 4.43 g/L; Sucrose: 30 g/L; Kinetin: 50 mg/L; Phytagel 3 g/L; pH: 5.8). While in the root development, media was also supplemented with the growth hormones IAA (1 mg/L) and IBA (1 mg/L). The plants were shifted to pots after proper development of shoots and roots. The soil mixture used in pots was of the same composition as described by Rao et al. [32]. When plants were able to tolerate 6 h sunlight, they were shifted to the field (Figs. 2 and 3). The seeds of *T*_0_ PCR-positive transgenic plants were used to rise *T*_1_ generation, while the seeds of confirmed transgenic cotton plants of *T*_1_ generation were then used to advance *T*_2_ generation.

### Molecular Analysis

#### Polymerase Chain Reaction (PCR)

The CTAB method was used for genomic DNA isolation of putative transgenic cotton plants [33]. Amplification of the transgene with specific primers was done by PCR to confirm the successful transformation. DNA isolated from putative plants was used as a template; the plasmid construct was used as positive control, while the DNA isolated from untransformed plants was used as negative control. The PCR master mix was composed of 3 µl (10 ng) of template DNA, 2 µl PCR Buffer (10×), 2.5 µl MgCl_2_, 2 µl forward primer (10 pmol), 2 µl reverse primer (10 pmol), 2µl dNTPs (2 mM), and 1 µl (1.25 units) of *Taq* DNA polymerase (Fermantas cat # EP0402). The PCR reaction was initiated with denaturation at 95 °C for 5 min and subjected to 35 cycles as follows: 95 °C for 1 min, 59 °C for 1 min, and 72 °C for 1 min. Extension phase was prolonged for 10 min at 72 °C. The transgenic plants were screened at *T*_0_, *T*_1_ and *T*_2_ generation on the basis of PCR results.

#### Monitoring of Disease Symptoms and Determination of Viral Disease Index

Monitoring of CLCuV symptoms of transgenic cotton plants was done through random selection. None of the plant was sprayed with whitefly control. The disease index was calculated using a scale described by Akhtar and Khan [34]. The inoculation of transgenic along with control cotton plant was done by incubating each transgenic and control plant with ten viruliferous whiteflies (produced by feeding on symptomatic non-transgenic plants). PCR reaction was performed to confirm and select the infected whiteflies in controlled greenhouse condition, and observation were taken after 3 week-interval of plant inoculation with whitefly.

Percentage disease index was established by following the procedure determined by Farooq et al. [35] and applied in cotton by Akhtar et al. [36]. According to this formula, disease index of cotton line with a factor 100/6 in which 100 determined percentage and 6 determined total level of disease index [35, 36].

$${\text{Percent Disease index}} = \frac{\text{Sum of all disease ratings of selected plants at random}}{{{\text{Total no}}.{\text{ of plants}}}} \times \frac{100}{6}.$$

#### Virus Titer Determination Through Real-Time Quantitative PCR

The virus titer in different transgenic plants with amplicon V2 RNAi was determined through Thermo Scientific Maxima SYBR Green qPCR kit (cat# K0241). The DNA of transgenic and non-transformed plants was diluted 10× before using as a template. CLCuKoV-Bur plasmid construct and CLCuMB plasmid construct were used as the standard for absolute quantification, while virus-infected plants were used as positive control. The master mix contained 10 µl SYBER green, 0.35 µl of Forward primer (10 pmol, Table 1), 0.35 µl of Reverse primer (10 pmol, Table 1), and 8.3 µl of template DNA (100/reaction). The qPCR reaction was started with an initial denaturation at 95 °C for 10 min, 40 cycles of 95 °C for 15 s, 59 °C for 30 s, and 72 °C for 30 s. A final extension was given at 72 °C for 10 min.

#### Southern Blot Analysis

The transgene copy no was determined using the Southern blot analysis as described by Southern [37]. Genomic DNA from apical leaves of putative transgenic cotton plants and untransformed control plants was isolated using Thermo scientific Genomic DNA purification kit (cat # K0512) by following the manufacturer’s guidelines. Genomic DNA (20 μg) was digested with HindIII enzyme according to the supplier’s instructions (Enzyme Production Lab of the National Centre of excellence in molecular biology (CEMB), Pakistan). The color was detected by 5-bromo-4-chloro-3-indolyl phosphate/nitro blue tetrazolium (BCIP/NBT) tablets (Sigma B5655) dissolved in water according to the manufacturer’s instruction.

#### Fluorescence In Situ Hybridization (FISH)

Labeling of the probe for transgene detection was done by Fluorescein ULS^®^ Labeling Kit (Fermentas K0641) by following the instructions given by the manufacturer, and in situ hybridization was carried out according to protocol described by Rahman et al. [38] on metaphase chromosomal spreads.

## Results

### Transformation of RNAi Gene Construct in Cotton Embryos

The *Agrobacterium* shoot apex method of transformation optimized at CEMB was used to transform MNH-786 and VH-28 varieties of cotton. Eighty-six putative transgenic plants were obtained from 43 transgenic experiments of MNH-786, while 43 putative transgenic plants were obtained from 41 transgenic experiments of VH-289 (Table 2).

**Table 2:** The record of transformation experiments done for RNAi construct transformation

Variety	Experiments	Total seeds	No. of embryos isolated	No. of shoots in test tubes after 30 days	Acclimatization		Plants acclimatized and thrived in field
					No. of plants shifted to soil pots	No. of plants shifted to Field	
MNH786	43	27,300	7400	278	86	24	**12**
VH289	41	26,700	6800	196	43	26	**11**

Bold values are the important facts and figures about results

The high mortality rates from embryo inoculation till the development of rooted plantlets on kanamycin selection media showed low transformation efficiency rates i.e., 3.75 % in the case of MNH-786 and 2.88 % in case of VH-289 (Table 3).

**Table 3:** Transformation Efficiency of *Agrobacterium*-mediated transformation

Variety	No. of embryos isolated	No. of plants obtained after 8 weeks	Transformation efficiency (%)
MNH786	7400	278	**3.75**
VH289	6800	196	**2.88**

Bold values are the important facts and figures about results

### Confirmation of Gene Integration in *T*_0_, *T*_1_, and *T*_2_ Transgenic Plants

**Fig. 4:**
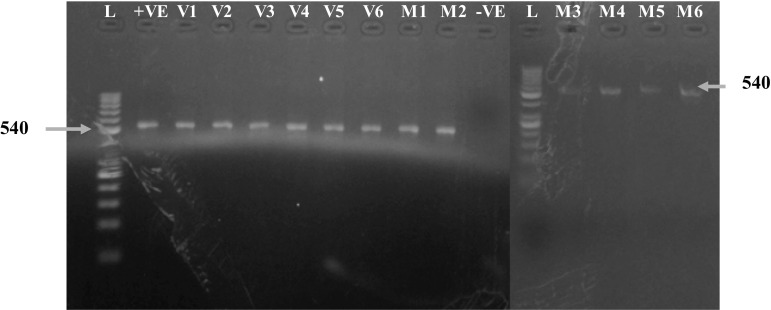
PCR analysis of putative transgenic plants (VH-289 & MNH-786) *L* 50 bp DNA ladder; −*ve* the non-transgenic plant, +*ve* plasmid construct was used as positive control, *V*1–*V*6 putative transgenic plants of VH-289, *M*1–*M*6 putative transgenic plants of MNH-786

PCR analysis was performed to confirm the integration of the gene construct in three generations of transgenic plants. Eighty-six putative transgenic cotton plants of MNH-786 and 43 putative transgenic cotton plants of VH-289 were analyzed. Confirmation of successful transformation of putative transgenic cotton plants was done by PCR using plasmid as positive control and wild-type cotton plants as negative controls. PCR analysis (Fig. 4) confirmed the successful transformation of the amplicon V2 RNAi construct in these newly transformed cotton lines. The amplification of 540 bp internal fragments of RNAi construct in 26 putative transgenic plants of MNH-786 and 26 putative transgenic plants of VH-289 was achieved. No amplification was detected in negative control.

A total of 11 MNH-786 and 12 VH-289 transgenic events were used for cultivation of the *T*_1_ generation. The plants were also analyzed. PCR to amplify a 540 bp region of amplicon V2 RNAi construct was performed for *T*_1_ transgenic cotton using plasmid as positive and wild-type cotton plants as negative controls with the same primers as were used for analysis of putative transgenic plants in *T*_0_ generation. Seeds of transgenic *T*_1_ events were used to rise the *T*_2_ generation. The *T*_2_ generation plants were tested through PCR. PCR analysis confirmed the presence of the amplicon V2 RNAi construct.

### Viral Disease Index

**Fig. 5:**
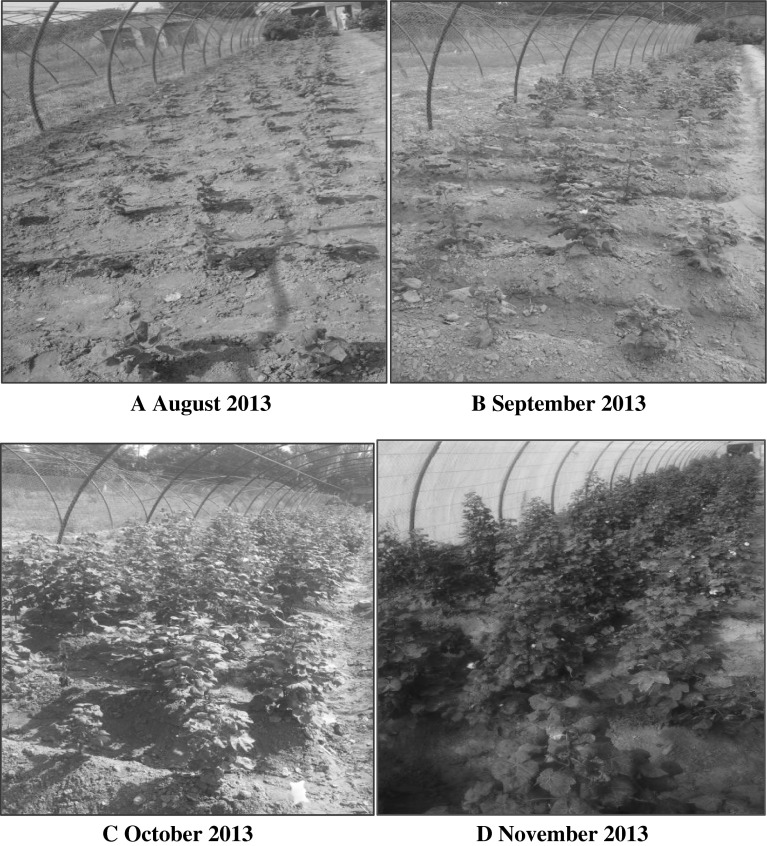
Pictorial View of Onset of CLCuV on T1 generation observed in different months. **a** Mild Symptoms of CLCuV on transgenic Cotton Plants. **b** Persistence of Mild Symptoms (CLCuV Tolerance) by Transgenic Cotton Plants. **c** Recovery of Plants vegetatively and reproductively while tolerating and minimizing viral titer. **d** Cotton Plants at Maturity showing tolerance of CLCuV with mild symptoms

The transgenic plants in the *T*_1_ generation were found to be affected by the CLCuD infection on August 2013. Continuous observations were made for four consecutive months, starting from onset of disease. Monthly pictorial view of *T*_1_ transgenic plants is shown in Fig. 5.

To calculate the viral disease index, each *T*_1_ transgenic plant was rated as per criteria described by Akhtar and Khan [34] (Table 4).

**Table 4:** Viral disease Index of transgenic plants

Symptoms	Disease ratings	Disease index (%)	Disease reaction
Absence of symptoms	0	0	Immune
Thickening of a few small veins or the presence of leaf enations on 10 or fewer leaves of plants	1	0.1–1	Highly resistant
Thickening of the small group of veins	2	1.1–5	Resistant
Thickening of veins, but no leaf curling	3	5.1–10	Moderately resistant
Severe vein thickening or leaf curling at the top of the third plant	4	10.1–15	Moderately susceptible
Severe vein thickening or leaf curling on half of the plant	5	15.1–20	Susceptible
Severe vein thickening, leaf curling, stunted growth of the plant and less fruit production	6	>20	Highly susceptible

Different plants showed different ratings. The plants with maximum and minimum disease ratings were selected for viral titer determination through real-time quantitative PCR (qPCR). From ten transgenic VH-289 and MNH-786 lines along with non transgenic control lines, total one hundrad plants were screened. The number of plants of each variety with their disease ratings is given in Table 5.

**Table 5:** Disease ratings of transgenic and non-transgenic plants

Sr. No	Plants types	No. of plants under disease rating 6	No. of plants under disease rating 5	No. of plants under disease rating 4	No. of plants under disease rating 3	No. of plants under disease rating 2	No. of plants under disease rating 1	No. of plants under disease rating 0
1	Non-transgenic control plants	30	21	19	30	0	0	0
2	Transgenic plants MNH-786	5	5	6	57	27	0	0
3	Transgenic plants MVH-289	2	10	18	36	34	0	0

### Virus Titer of *T*_1_ Transgenic Plants

**Fig. 6:**
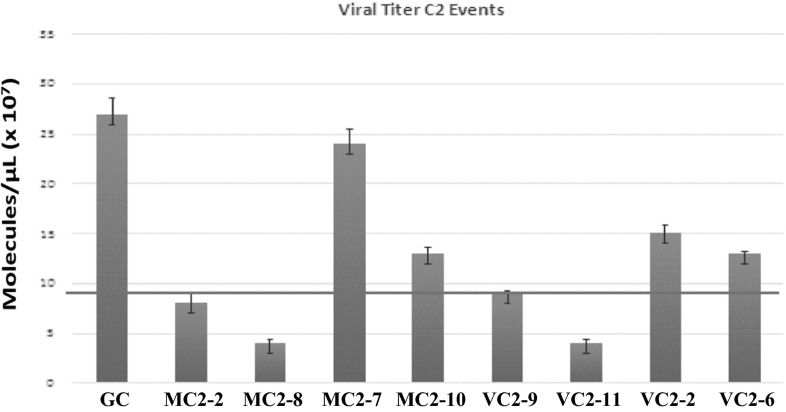
Viral Titer calculation via real-Time PCR in T1 transgenic plants *GC*2 −ve control, *MC*2-2, *MC*2-8, *MC*2-7, and *MC*2-10 Transgenic plants of MNH-786; *VC*2-9, *VC*2-11, *VC*2-2, and *VC*2-6 transgenic plants of VH-289 in T1 generation harboring RNAi gene construct

Absolute quantification via qPCR was used to determine the virus titer in eight different events of *T*_1_ generation. *A wild-type plant was used as negative control. It is* obvious from Fig. 6 that all selected events showed a different virus titer. A tentative demarcation line was inserted (shown in green) to select the plants with minimum viral titer. The results were quite promising as the plants with lower disease index rating, i.e., MC2-2, MC2-8, VC2-9, and VC2-11 also showed low virus titer in qPCR.

**Fig. 7:**
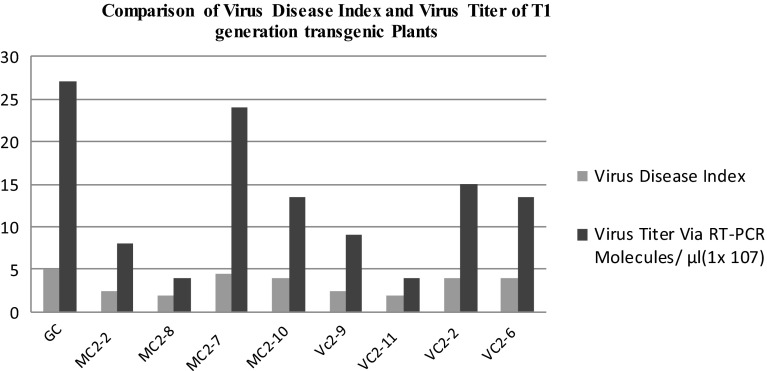
Comparison of viral disease index and virus titer of T1 generation transgenic Plants. *GC* non-transgenic plant, *MC*2-2, *MC*2-8, *MC*2-7, and *MC*2-10 Transgenic plants of MNH-786, *VC*2-9, *VC*2-11, *VC*2-2, and *VC*2-6 transgenic plants of VH-289

The results of the CLCuD disease index and viral titer determined via qPCR were compared as shown in Fig. 7. It showed that symptom severity was directly proportional to virus titer. Plants having high virus titer exhibited more severe symptoms than those plants having low virus titer.

**Fig. 8:**
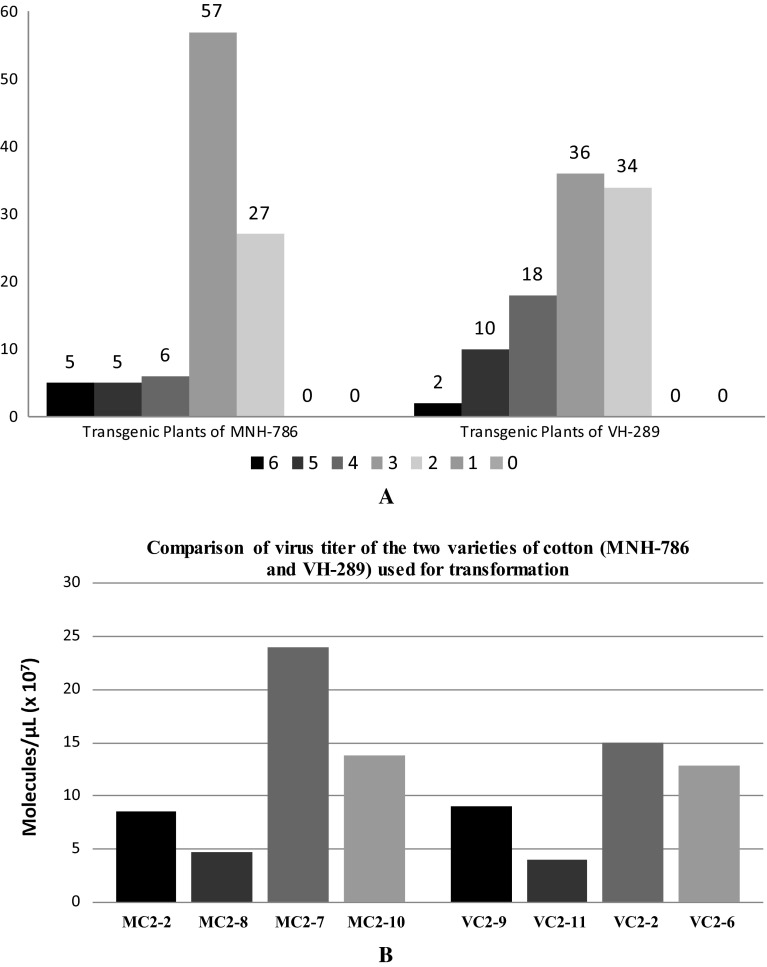
**a** Comparison of disease index of the two varieties of cotton (MNH-786 and VH-289) used for transformation. *Y*-axis is showing number of plants, while *X*-axis is showing different disease ratings (0–6) as per Table 4. **b** Comparison of virus titer of the two varieties of cotton (MNH-786 and VH-289) used for transformation. *Y*-axis is showing virus titer in terms of molecules/µl × 10^7^, while *X*-axis is showing transgenic plants, MC2-2, MC2-8, MC2-7, and MC2-10 of MNH-786 and VC2-9, VC2-11, VC2-2, and VC2-6 of VH-289

A comparative study of virus titer and virus disease index of both transgenic varieties was also performed (Fig. 8a, b).

### Location of Transgene in Cotton

#### Southern Blot Analysis

**Fig. 9:**
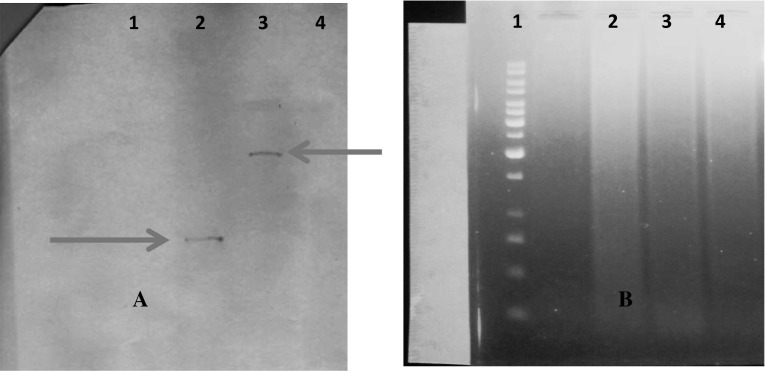
**a** and **b** Southern Blot Analysis and Restriction digestion gel of Southern for RNAi in transgenic Cotton plant *Lane 1* 1 Kb DNA Ladder, *Lane 2* MC2-8B transgenic cotton plant of MNH 786 variety, *Lane 3* VC2-11 transgenic cotton plant of variety VH-289, *Lane 4* Non-transgenic cotton control plant

The stable integration of RNAi in the cotton plant genome and transgene copy number was confirmed by the Southern blot analysis. The copy number of RNAi transgene was obtained by a specific probe, which highlighted a different copy number based on restriction digestion of genomic DNA with unique sites using HindIII enzyme. The results clearly depict one copy number in transgenic cotton plants VC2-11 and MC2-8B respectively (Fig. 9).

### Fluorescence In Situ Hybridization

**Fig. 10:**
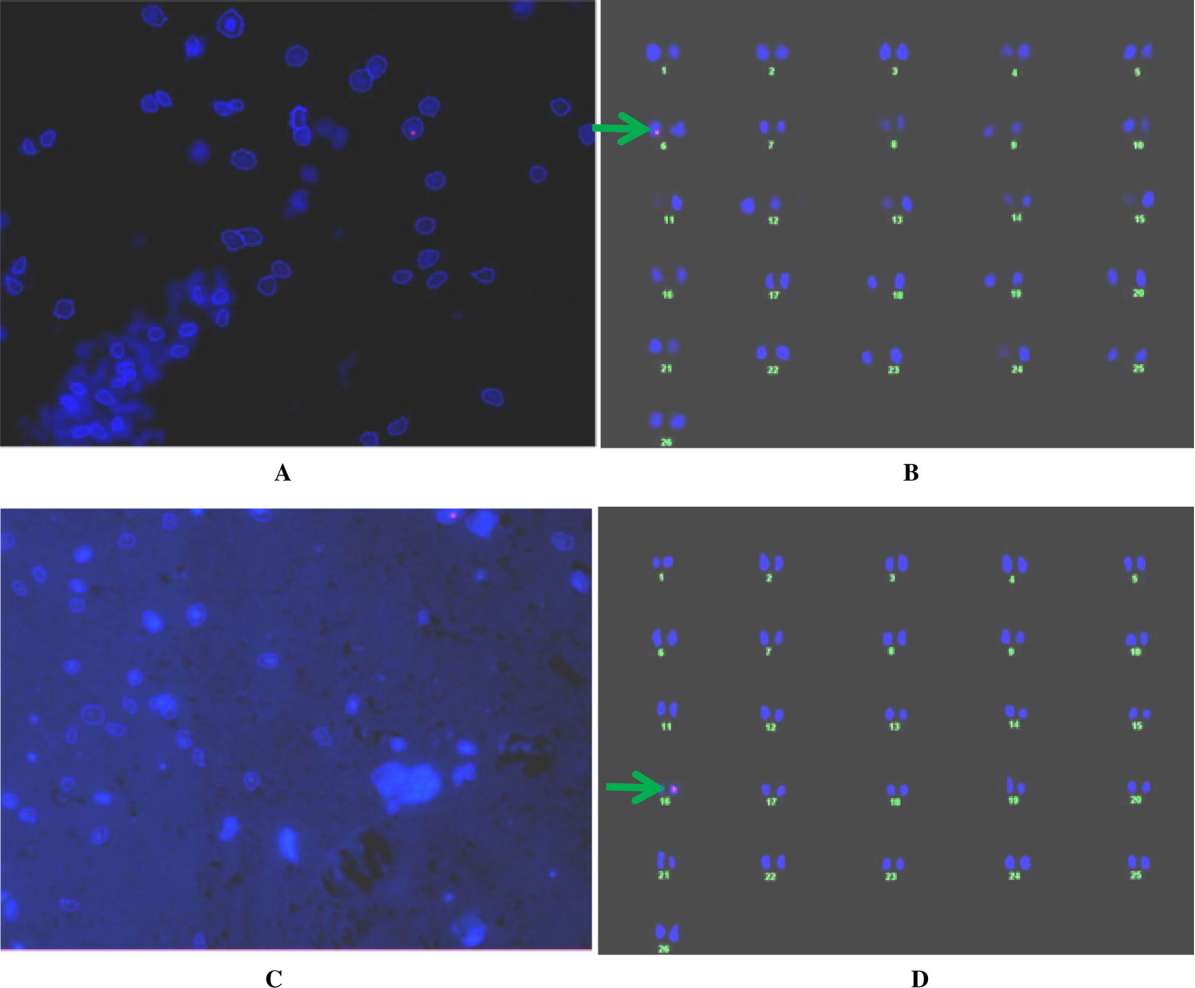
**a**, **b**, **c,** and **d** Fluorescence in situ hybridization (FISH) of RNAi of two different plants Construct 2 i.e., MC2-8B (**a** and **b**) and VC2-11 (**c** and **d**). **a** Metastatic data of MC2-8B transgenic plant, **b** Karyotping of RNAi transgene of transgenic plant MC2-8B transgenic plant, **c** Metastatic data of VC2-11 transgenic plant, **d** Karyotping of RNAi transgene of transgenic plant VC2-11 transgenic plant

Integration of amplicon V2 RNAi in cotton plants was also confirmed by fluorescent in situ hybridization. There was random integration of the RNAi gene in Chromosome 6 and 16. One copy at Chromosome 6 was observed in transgenic event MC2-8 from the MNH 796 variety, while a single copy at chromosome 16 was observed in transgenic event VC2-11 from variety VH-289 (Fig. 10a–d).

## Discussion

Cotton is the white gold of Pakistan’s economy. Pakistan is the largest exporter of cotton yarn. Therefore the efforts to improve crop production, improve fiber quality, and pest management by genetic modification are critical. Cultivation of resistant crops and use of insecticides are currently the two major controls used for CLCuD [39]. According to Mansoor et al. [5], CLCuD-resistant crops were successfully developed in 1990s, but the virus soon developed resistance to these varieties [40]. An alternative strategy to control CLCuD could be RNAi. The results presented here have shown that the amplicon V2 RNAi approach, which is targeted against highly conserved V2 gene of the begomovirus, has the potential to provide resistance against CLCuKoV-Bur CLCuD in transgenic cotton plants. The majority of the transgenic cotton plants, upon challenge with viruliferous whiteflies in a contained environment, showed lower disease symptoms as well as disease index ratings. qPCR analyses showed the presence of virus in all transgenic plants, but there was a significant difference in virus titer compared with wild-type cotton plants. The level of resistance obtained here may be more accurately stated as highly tolerant to infection. These results are consistent with several studies that have exploited the RNAi approach for transgenic resistance against geminiviruses. According to Ali et al. [43], effective resistance in plants against monopartite begomoviruses can be obtained by applying a miRNA approach. [15, 41–44].

In the present study, the amplicon V2 RNAi gene construct was transformed in elite cotton varieties MNH-786 and VH-289. V2 protein in monopartite virus is a symptom determinant and elicited cell death. When expressed in plants using *Potato virus X*-based vector, it can act as a suppressor of gene silencing [45, 46]. Moreover, V2 encoded by CLCuMV is a very strong suppressor of gene silencing [10]. Tobacco plants were developed harboring an antisense construct targeting the AV2 gene of *Tomato leaf curl New Delhi Virus* (ToLCNDV) [47]. Upon challenging with virus-infectious clones of ToLCNDV, transgenic plants remained asymptomatic, although viral DNA could be detected by PCR. Satyavathi et al. [29] transformed Indian cotton variety F-846 with *Agrobacterium* transformation by targeting V2 genes of CLCuKoV using a RNAi approach [29]. Transgenic cotton plants exhibited a true Mendelian pattern of inheritance and were tolerant to complex virus including CLCuD. In this study, we are not only reporting the development of cotton transgenic plants, but we have conducted a comprehensive study on the resistance of amplicon V2 RNAi construct. Our results have shown that the amplicon V2 RNAi approach can provide high tolerance in transgenic cotton plants. In order to achieve a resistance to immunity, the amplicon V2 RNAi can be coupled with other types of resistance and tolerance.

Cotton varieties MNH-786 and VH-289 were transformed via *Agrobacterium* Strain LBA4404. A total of 84 experiments were performed to transform the RNAi gene in cotton. The number of plantlets obtained after 8 weeks for both transgenic varieties was low, i.e., 278 for MNH-786 and 196 for VH-289. Thus, the transformation efficiency of MNH-786 and VH-289 was 3.75 % and 2.88 %, respectively (Table 3). Bakhsh et al. [48] reported higher transformation efficiency (20 %) of *Agrobacterium* strain (LBA4404) in tobacco plants [48], but cotton is harder to transform, and our results were similar to the work of Majeed et al. [49] in which transformation efficiency in cotton was 5.17 % [49, 50].

Eighty-six putative transgenic plants of MNH-786 and 43 putative transgenic rooted plants of VH-289 were shifted to soil pots. PCR confirmed the successful integration of the RNAi gene into cotton genome. The number of plants was further reduced during acclimatization, and the numbers of T0 generation plants shifted to the field was 24 and 26 for MNH-786 and VH-289, respectively. Once the plants began flowering, their flowers were self-pollinated to avoid gene transfer [51].

The seeds of the T_0_ generation were sown in the field during August 2013, with ten seeds of a single event sown in a row. The plants were not protected from whitefly attack at this stage.

The whitefly attack on transgenic plants of the *T*_1_ generation was seen at a very early stage of sowing. The plants were continuously observed during this menace. The viral index of all plants in the field was calculated using a formula as described by Akhtar and Khan [34]. Four plants were selected from both varieties (VH-289 and MNH-786) along with one non-transgenic plant to determine the viral titer via RT-PCR. The plants having minimum symptoms showed minimum viral titer and vice versa (Fig. 7). The results of real-time PCR were of utmost importance for the selection of plants for the *T*_2_ generation. The other important factor considered for selection of plants was the PCR result which confirmed RNAi gene integration in *T*_1_ plants. The integration of RNAi gene construct in plants of *T*_2_ generation was confirmed via PCR using gene-specific internal primers. Three out of six MNH-786 and five out of eight VH-289 T3 plants were confirmed after PCR analysis. qPCR assay was performed to quantify virus titer in plants showing minimum and maximum symptoms (from each variety). Interestingly, a positive correlation was observed between the virus titer and disease index.

Both the varieties (MNH-786 and VH-289) showed almost similar results.

To determine the transgene location and copy number in the transgenic plants, the FISH analysis was done. Transformants with a single copy were achieved in both the varieties. In the case of two events from different varieties, the transgene locations were observed on different chromosomes. Transformed plants (vv. MC2-8) showed transgene insertion at chromosome No. 6, whereas in VC2-11 line, it was at chromosome No. 16. This has been observed in other transformation events and the different locations maybe due to various factors which are involved in the transformation of the transgene into the host genome [52, 53].

## Conclusion

The molecular analyses performed in this study showed the positive integration of the RNAi gene construct in *T*_0_, *T*_1_, and *T*_2_ generations. Cotton is an important crop not only for Pakistan, but also India where CLCuD is a big threat to cotton as well. Any successful transgenic varieties could have a big impact in countries throughout the globe where CLCuD is a threat. The positive integration of an RNAi-based gene in cotton varieties and absolute quantification determining viral titer of transgenic plants gives hope for a promising future for transgenic crops in Pakistan.
